# Data on the viscoelastic behavior of neoprene rubber

**DOI:** 10.1016/j.dib.2018.10.081

**Published:** 2018-10-27

**Authors:** Deepak Kumar, Somnath Sarangi

**Affiliations:** Department of Mechanical Engineering, Indian Institute of Technology Patna, Bihta 801103, India

## Abstract

The present article contains data on the multi-step cyclic stress relaxation tests associated with the viscoelastic behavior of the neoprene rubber. Herein, the present data aims the accurate prediction of the time dependent mechanical behavior of the polymeric materials. The findings of the present data include the demonstration of the Mullin׳s stress-softening phenomenon, clearly. These data findings may serve as a benchmark to validate the more advanced phenomenological model developments in future as compared to the existing ones.

**Specifications table**

**Table:** 

Subject area	*Materials Science*
More specific subject area	*Polymeric Materials*
Type of data	*Graph, Figure*
How data was acquired	*Tinius Olsen H5KS Universal Testing Machine*
Data format	*Raw*
Experimental factors	*Four dumbbells specimens of the rubber were made according to ASTM specification D638-10 (Type-I) (ASTM-D638, 2010)*
Experimental features	*A neoprene rubber material was used for the multi-step cyclic stress relaxation tests. The experimental tests were conducted at room temperature.*
Data source location	*Department of Mechanical Engineering, Indian Institute of Technology Patna, Bihar, India*
Data accessibility	*The data are with the related research article*[4].
Related research article	*Laiarinandrasana, L., R. Piques, and A. Robisson. "Visco-hyperelastic model with internal state variable coupled with discontinuous damage concept under total Lagrangian formulation." International Journal of Plasticity 19.7 (2003): 977–1000.*[3]

**Value of the data**

• The obtained multi-step cyclic stress relaxation test data are a good candidate to validate the viscoelastic behavior of neoprene rubber through the constitutive modeling of the rubber-like materials.
• The findings of the data may serve as a benchmark to validate the more advanced phenomenological model developments in future as compared to the existing ones.
• These data also add the potential value in characterizing physical mechanisms of the polymeric rubbery materials in future.

## 1 Data

Attaining the multistep-stress relaxation test data is a classical way to model the time-dependent behaviour of the soft materials, and it allows to access different deformation phenomena too [1], [2]. Herein, the experimental data include the multi-step cyclic stress relaxation tests in appropriate details. These details contain four different tests with the corresponding strain rates and relaxation time as shown in the Table 1. We designed four multi-step cyclic stress relaxation tests to investigate the time-dependent behavior of neoprene rubber.

**Table 1:** Experimental-tests detail.

**Multi-step cyclic stress relaxation tests**	**Strain-rate (s**^**-1**^**)**	**Relaxation time (s)**
**Test-1**	0.01	20
**Test-2**	0.01	40
**Test-3**	0.02	40
**Test-4**	0.04	40

## 2 Experimental design, materials, and methods

### 2.1 Specimen detail

For the data collection, a neoprene rubber material was used for the multi-step cyclic stress relaxation tests. Four dumbbells specimens of neoprene rubber were made according to ASTM specification D638-10 (Type-I) (ASTM-D638, 2010), and the experiment is conducted at room temperature. The standard gage length of the test specimen was 50 mm as shown in Fig. 5.

**Fig. 1:**
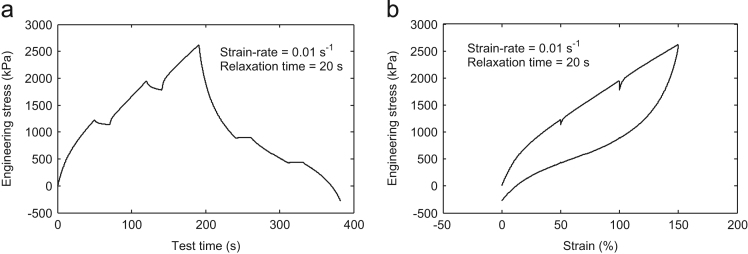
Multi-step cyclic stress relaxation **Test-1** at 0.01 s^-1^ strain-rate and 20 s relaxation time.

**Fig. 2:**
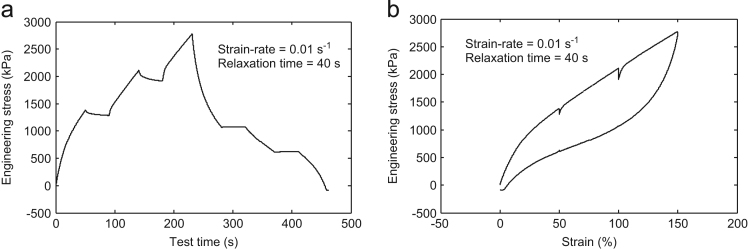
Multi-step cyclic stress relaxation **Test-2** at 0.01 s^-1^ strain-rate and 40 s relaxation time.

**Fig. 3:**
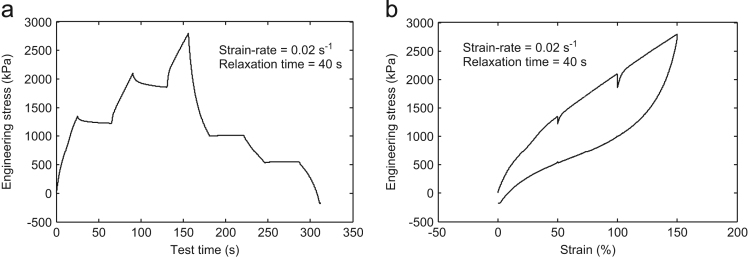
Multi-step cyclic stress relaxation **Test-3** at 0.02 s^-1^ strain-rate and 40 s relaxation time.

**Fig. 4:**
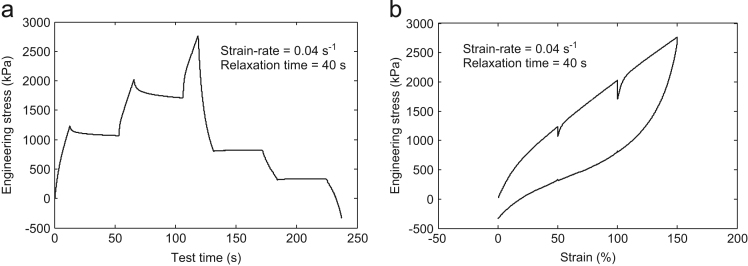
Multi-step cyclic stress relaxation **Test-4** at 0.04 s^-1^ strain-rate and 40 s relaxation time.

**Fig. 5:**
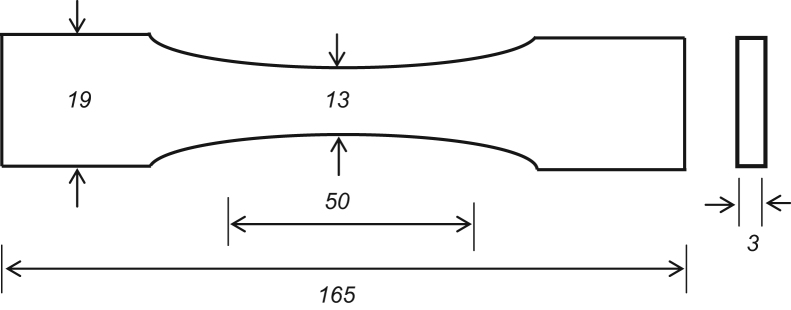
Standard test specimen (all the dimensions are in mm).

### 2.2 Machine detail

For the data collection, Tinius Olsen H5KS universal testing machine with 250 N load cell capacity was used to test the viscoelastic property of the neoprene rubber.

### 2.3 Working method

For the data collection, the strain steps were taken as 50%, 100%, and 150% in all the tests. In the **Test-**1, the specimen was first strained up to 50% at a strain rate of 0.01 s^-1^ and hold there for 20 s. Then, it was again strained up to 100% and was held there for predefined relaxation period 20 s. Further, it was again strained up to the final value of 150%. This completes the half cycle of loading. Now, for the unloading cycle, the whole process was repeated immediately at the predefined strain rate of 0.01 s^-1^ and 20 s relaxation time. The same experimental procedure was performed for the other tests also with the corresponding strain rate and the relaxation time shown in Table 1. The other tests detail may be obtained from the output plots as shown in Fig. 1, Fig. 2, Fig. 3, Fig. 4.

### 2.4 Data applications

The collected data on the multistep-cyclic stress relaxation tests for a neoprene rubber get applications in the field of soft material modeling, which may help to enhance the accuracy of the time dependent behavior.
