# Supplementary material for: Data on the viscoelastic behavior of neoprene rubber
Source: Data Brief. 2018 Oct 27;21:943–7. doi: 10.1016/j.dib.2018.10.081 (PMC6222071; doi:10.1016/j.dib.2018.10.081)
Supplement: Supplementary file 1 — Supplementary material [file mmc1.pdf]

Cover Letter/Conflicts of Interest

October 18, 2018

Editorial Department of Data in Brief

Respected Editor of Data in Brief,

I am submitting a manuscript for consideration of publication in Department of Data in Brief. The manuscript is entitled “**Data on the viscoelastic behavior of neoprene rubber**”. It has not been published elsewhere, and that it has not been submitted simultaneously for publication elsewhere.

Thank you very much for your consideration.

Yours Sincerely,

Deepak Kumar

Indian Institute of Technology Patna

Dynamics & Vibration Lab, IIT Patna, Bihta, India-801103

Tel.: +91-8294918852;

E-mail: dkumar.pme14@iitp.ac.in
